# Complexity Level Analysis Revisited: What Can 30 Years of Hindsight Tell Us about How the Brain Might Represent Visual Information?

**DOI:** 10.3389/fpsyg.2017.01216

**Published:** 2017-08-09

**Authors:** John K. Tsotsos

**Affiliations:** Department of Electrical Engineering and Computer Science, York University Toronto, ON, Canada

**Keywords:** vision, attention, complexity, pyramid representations, selective tuning model

## Abstract

Much has been written about how the biological brain might represent and process visual information, and how this might inspire and inform machine vision systems. Indeed, tremendous progress has been made, and especially during the last decade in the latter area. However, a key question seems too often, if not mostly, be ignored. This question is simply: do proposed solutions scale with the reality of the brain's resources? This scaling question applies equally to brain and to machine solutions. A number of papers have examined the inherent computational difficulty of visual information processing using theoretical and empirical methods. The main goal of this activity had three components: to understand the deep nature of the computational problem of visual information processing; to discover how well the computational difficulty of vision matches to the fixed resources of biological seeing systems; and, to abstract from the matching exercise the key principles that lead to the observed characteristics of biological visual performance. This set of components was termed *complexity level analysis* in Tsotsos (1987) and was proposed as an important complement to Marr's three levels of analysis. This paper revisits that work with the advantage that decades of hindsight can provide.

## Introduction

This paper has two main parts. In the first, there is a brief recapitulation of 30 years of research^1^ that addresses the question: do proposed solutions to how the brain processes visual information match the reality of the brain's resources? The main goal of this activity had three components: to understand the deep nature of the computational problem of visual information processing; to discover how well the computational difficulty of vision matches to the fixed resources of biological seeing systems; and, to abstract from the matching exercise the key principles that lead to the observed characteristics of biological visual performance. The second part of the paper uses the results of that analysis and extends them to specifically connect to how the brain represents visual information. We begin by motivating the analysis as presented three decades ago.

A universally acclaimed landmark in the development of computational theories of intelligence is the presentation of the three levels of analysis defined by Marr (1982). Marr presents the three levels, now quoted, at which any machine carrying out an information-processing task must be understood:

- *Computational theory*: What is the goal of the computation, why is it appropriate, and what is the logic of the strategy by which it can be carried out?
- *Representation and algorithm*: How can this computational theory be implemented? In particular, what is the representation for the input and output, and what is the algorithm for the transformation?
- *Hardware implementation*: How can the representation and algorithm be realized physically?

This prescription has been used effectively ever since not only in vision modeling but throughout computational neuroscience and cognitive science. Unfortunately, Marr, not being a computer scientist, missed an important issue. He did not realize that it is not difficult to pose perfectly sensible computational solutions that are physically unrealizable. As argued in Tsotsos (1990) and elsewhere, there are a large number of perfectly well-defined computational problems whose general solution is provably intractable—unrealizable on available physical resources or requiring time longer than the age of the universe^2^. Even worse, there are well-defined problems that are undecidable, meaning there provably exists no algorithm to determine the result^3^. As argued in Tsotsos (1993, 2011), such results that seem impossible do not negate their main impact: our brains seem to deal with all the problems they face remarkably well so it can only be the case that the formal definitions of the problems that lead to such intractable or impossible results cannot be the ones that our brains are actually solving.

This matching process as an idea has its roots in earlier works. Uhr (1972, 1975) describes “recognition cones” as a representation for perception. Although his papers are clear in their inspiration from neural systems, Uhr only hinted at their resource implications. Feldman and Ballard (1982), however, explicitly linked computational complexity to neural processes saying “Contemporary computer science has sharpened our notions of what is ‘computable’ to include bounds on time, storage, and other resources. It does not seem unreasonable to require that computational models in cognitive science be at least plausible in their postulated resource requirements.” They go on to examine the resources of time and numbers of processors, and more, leading to a key conclusion that complex behaviors can be carried out in fewer than 100 (neural processing) time steps. The overall import of their paper was to stress the need for a careful matching of problem to resources in cognitive theories. *Resource-complexity matching is a source of critical constraints on the viability of theories*, especially those that attempt to provide a mechanistic theory as opposed to a descriptive one (see Brown, 2014).

Even though these arguments were very strong, they took the form of ‘counting arguments’ and a formalization could perhaps make them even stronger. An attempt to formalize those points was made beginning with Tsotsos (1987). We examined the inherent computational difficulty of visual information processing from formal and empirical perspectives^4^. The methods used have their roots in the theoretical sub-domain of computer science known as computational complexity. Computational complexity has the goal of discovering formal characterizations of the difficulty of achieving solutions to computational problems^5^ in terms of the size and nature of the input. The difficulty of achieving solutions has direct impact on resources, such as computational power, memory capacity and processing time, as Feldman and Ballard (1982) also pointed out.

For this reason, a fourth level, the complexity level, was introduced in Tsotsos (1987, 1990), intended to ensure the logic of the strategy for solving the problem is actually realizable within its available resources:

- *Complexity analysis:* What is the computational complexity of the problem being addressed? How does it match with the resources used for its realization? If the problem is intractable and/or there are insufficient resources available for a realization of its solution, how can the problem be reframed to enable a solution?

This paper revisits the conclusions reached by the resulting series of papers with the advantage of decades of hindsight. Interestingly, a wide spectrum of predictions regarding the brain's visual processes that resulted from that analysis has enjoyed subsequent experimental support (see Tsotsos, 2011 for details). We begin with a brief overview of the main conclusions and assertions that complexity level analysis provided.

## Complexity level analysis

In Tsotsos (1989, 2011), a number of mathematical proofs were presented that formalize the difficulty of perhaps the most elemental of visual operations—essentially a sub-element of all visual operations—namely, visual matching^6^. Visual matching is the task of determining if some arbitrary image, a goal image, is a subset of some other image, the test image. In this definition, no knowledge of the target is allowed to influence the solution—the problem is thus termed *unbounded* in those papers. A function was assumed to exist that would quickly determine if a particular match was found, and it was not permitted to reverse engineer that function in order to guide the search. In other words, the solution was constrained to be one requiring a strictly data-driven approach^7^. The main proof, replicated by Rensink (1989) using a different approach, showed that this problem potentially had exponential time complexity in the number of image pixels, largely because in the worst case, it is unknown which image subset is the one that represents that goal image (think of an arbitrary sky full of stars—which subset of stars forms a hexagon?). The more important part of this is that it was proved that no single solution exists that is optimal for all possible problem instances. Due to the particular manner in which the proof was executed, the problem lends itself to a number of non-exponential, but not necessarily exact or optimal, solutions, as pointed out by Kube (1991)^8^. Following a more detailed examination, it was shown that although these non-exponential solutions are indeed valid, they do not really help because they all rely on solution elements that have no biological counterpart and have execution times that do not reflect human performance (Tsotsos, 1991)^9^. Note that this is likely true also for the other problems cited throughout this paper; they may also have known non-exponential solutions and realizable solutions for small enough or special case instances. A puzzling situation thus results: can we or can we not rely on the theoretical work as a guide? Our everyday experience with our own visual systems exhibits no such intractability. The only conclusion therefore is that the brain is not solving the problem as formalized for those proofs: the human brain is solving a different version of visual matching. This is admittedly a non-standard use of complexity theory because it disallows solutions that are not biologically realizable or plausible^10^. It does however show that the prevailing thoughts of the time (i.e., 1980's and somewhat beyond) that vision can be formulated as a purely bottom-up (i.e., stimulus-driven) process needed to be re-considered. To preview the endgame of this paper, that reformulation is one that allows differing levels of solution precision and different expenditures of processing time for different subsets of problem instances.

At this point in this presentation, it seems important to emphasize that the proofs mentioned in the previous paragraph do indeed point to sensible conclusions because there are many other researchers who have reached similar conclusions, i.e., that their problems are likely intractable, for a variety of visual and non-visual problems that are associated with human intelligent behavior. Selected examples of other works focusing on vision and neural networks and thus relevant for this paper include: polyhedral scene line-labeling (Kirousis and Papadimitriou, 1988); loading shallow architectures (neural network learning with finite depth networks) (Judd, 1988); relaxation procedures for constraint satisfaction networks (Kasif, 1990); finding a single, valid interpretation of a scene with occlusion (Cooper, 1998); unbounded stimulus-behavior search (Tsotsos, 1995a); and 3D sensor planning for visual search (Ye and Tsotsos, 1996).

The impact of computational complexity has also been pursued by many researchers in artificial intelligence and cognitive science (too many to properly mention here, however, see van Rooij, 2008, for a nice review). To round out this section, the important paper focusing on algorithm complexity, as opposed to problem complexity addressed by the previously cited authors, in vision by Grimson (1990) must be highlighted. Biologists also contributed with consistent and complementary conclusions (Thorpe and Imbert, 1989; Lennie, 2003, and others).

So how to proceed with the complexity level analysis? The whole point was to ensure that solutions are tractable within the constraints of biological processing structures. The strategy we chose which first appeared in Tsotsos (1987) is to simply start with the obvious, brute-force, worst-case complexity for the visual problem first described in this section's opening paragraph, termed Visual Match in Tsotsos (1989) and Comparison in Macmillan and Creelman (2005) (which is not provable as a bound on the time complexity in any way) and see how it might be altered to fit within a brain^11^. It's as if we were tasked, in some imaginary world, to design the first ever visual system from scratch. Tsotsos (2011) gives this simple-minded worst-case complexity as *O(P*^2^*2*^*P*^*2*^*M*^*)*^12^. *P* represents the number of image elements (pixels, photoreceptors), *M* is the number of features represented (e.g., color, shape, texture, etc.); these are the starting elements from which we need to design vision. Recall that the problem is termed ‘unbounded’ since there is no bounding information arising from task or world knowledge that limits the search—as designers of the first ever visual system, it might not yet be apparent to us that we need task or world knowledge! In other words, we begin with the Marr approach (see footnote 7). Any image subset can be the correct one, and thus the powerset of image elements gives the worst-case scenario, and processing proceeds in a purely data-directed manner. The three elements of the complexity function arise in the following manner: *P*^2^-the worst-case cost of computing the matching functions; *2*^*P*^-the worst-case number of image subsets in an image of P pixels; *2*^*M*^-the worst-case number of feature subsets associated with each pixel.

In Artificial Intelligence, a central concept is that of Rational Action. Rational Action, carried out by a rational agent, maximizes goal achievement given the agent's current knowledge, the agent's ability to acquire new knowledge, and the current computational and time resources available to the agent (Russell et al., 2003). In everyday behavior, we humans only rarely attempt to optimize solutions, but rather, just need to get something done (when drinking from a glass, we do not optimize the path to minimize energy or distance; rather, we simply want to get the glass to our mouth). In other words, we mostly resort to solutions that may not be optimal in any way but that are *good enough* for the current needs. Often, these are heuristic solutions that simply accomplish our goals^13^. One of these heuristics is to seek a *Satisficing* solution. Satisficing is a strategy that entails searching through the available alternatives until an acceptability threshold is met. This differs from *optimal decision-making*, an approach that attempts to find the best feasible alternative. The term *satisficing*, (a combination of *satisfy* and *suffice*), was introduced by Herb Simon in 1956. Satisficing can take more than one form. If one is faced with a problem and has the luxury of time, then one can spend as much time as one likes to find an acceptable solution among all the possible ones. One the other hand, if time is limited, perhaps strictly limited by the need to act before something else occurs, then a different sort of search would occur, one that would find a *just in time* solution, the best one within the time limit. If time is extremely tight, then an almost *reflexive* response is needed, perhaps the first one that comes to mind. Clearly, external tasks and situations as well as internal motivations play an important role in determining the right sort of approach to employ. Different from this strategy is the one where subsets of the full problem are defined where optimal procedures apply without infeasible characteristics. Here, the first step is to determine when such a problem is presented. Then, the most appropriate solution can be deployed. A rational agent, then, attempts to achieve its current goal, given its current constraints, by applying such selection methods to choose among its many possible solution paths. This points to the need for some kind of executive to control the process (one review for executive function in the brain, of the many available, can be found in Funahashi, 2001).

Knowledge of the intractability of visual processing in the general case—that is, that no single solution can be found that is optimal and realizable for all instances—forces a reframing of the original problem. The space of all problem instances can be partitioned into sub-spaces where each may be solvable by a different method. Some of those methods—whether satisficing, optimal, just in time, reflexive or other type—may lead to fast realizations (for example, if there is a special case problem subset that leads to non-exponential algorithm^14^), others slow ones, and some perhaps no realization. Given that a fixed processing resource such as the brain is to be employed, the need to apply a variety of different solution strategies in a situation dependent manner implies that resources must be *dynamically tunable*^15^. In order to support such a decision process, representations of visual, task, and world information and more must be available to support the reasoning involved that an executive controller performs (a sketch of how this might occur appears in Tsotsos and Womelsdorf, 2016).

**Figure 1:**
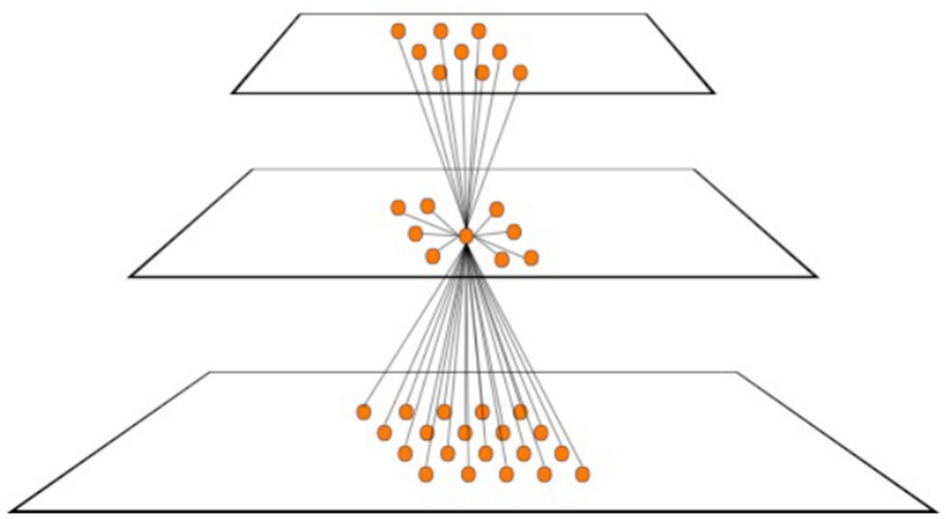
A hypothetical 3-layer pyramid representation. The number of locations represented in the bottom layer (layer 1) is *p*_*1*_*; p*_*1*_ > *p*_*2*_ > *p*_*3*_. A typical element of each layer is shown in the center of the middle layer (layer 2). The figure shows how that element is connected to its immediate neighbors in the layer, as well as to elements in the lower and higher layers. All connections are potentially bidirectional. The figure shows the converging pattern of feedforward connections from layer 1 to 2, the diverging pattern of feedforward connections from layer 2 to 3, the converging pattern of feedback connections from layer 3 to 2 and the diverging pattern of feedback connections from layer 2 to 1. Each element of each layer features this pattern of connectivity.

The second stage of complexity level analysis looks for ways of matching the available resources with the computational difficulty of the problem to be solved. For vision, and specifically for human vision, those resource constraints include numbers of neurons, synapses, neural transmission times, behavioral response times, and so on. As Garey and Johnson (1979) point out, using the main variables of the problem definition as a guide is useful; variables that appear in exponents are the most important to try and reduce. Only the conclusion of this exercise will be given here since the details have appeared in several past papers (see Tsotsos, 2011 for overview). The key activity is to reduce the worst-case time complexity expression so that it can lead to an algorithm that is matched to the size and behavior of the human brain. The main conclusions are:

1. Use a pyramid representation to reduce the number of image locations searched. A pyramid is a layered representation, each layer with decreasing spatial resolution and with bidirectional connections between locations in adjacent layers (Jolion and Rosenfeld, 1994 provide review). Introduced by Uhr (1972), they permit an image to be abstracted so that a smaller number of locations at the top level may be the only ones over which some algorithm needs to search. At least, they may provide the starting point for a coarse-to-fine search strategy from top to bottom of the pyramid. such a representation would reduce the size of the variable *P*. Figure 1 shows a hypothetical pyramid of 3 layers. The number of locations represented in the lowest layer (layer 1) is *p1*; *p1* > *p2* > *p3*. In most pyramid definitions, the value at each location in each layer is determined by a computation based on a subset of the other layer values. Each element is not only connected to others in the adjacent layers but may also be connected to elements within the same layer. Such a representation has much in common with the hierarchical organization of early visual cortex as revealed by the work of Hubel and Wiesel (1962, 1965).
2. The objects and events of the visual world are mostly spatially and temporally confined to some region; however, we can also recognize scattered items as well (such as star constellations, or collections of animals as flocks or herds, group motion say as in a rugby play, etc.). Spatio-temporally localized receptive fields reduce the number of possible receptive fields from *O(2*^*P*^*)* to *O(P*^1.5^*)* (this assumes contiguous receptive fields of all possible sizes centered at all locations in the image array and is derived in Tsotsos, 1987). Figure 1 not only shows a three-layer pyramid but also a typical element (neuron) within the middle layer and an illustration of the breadth of its connections within the pyramid showing that connectivity is limited in feedforward, feedback and lateral directions.
3. Selection of a single or group of receptive fields to consider can further reduce the *P*^1.5^ term to some value *P*′ < *P*^1.5^. This may be not only a selection of location, but also a selection of a local region or size. Such selection of region of interest is the most common use of attention in models (Tsotsos and Rothenstein, 2011; Tsotsos et al., 2015).
4. For some given task, feature selectivity to relevant features can further reduce the M term to some value *M*′, where 2^*M*′^ < 2^*M*^, that is, the subset *M*′ of all possible features actually present in the image or important for the task at hand. *M* ≪ *P* in any case so its presence in the exponent poses much less of a problem. This implies that features are best organized into separate representations, one for each feature, permitting a processing mechanism to involve only the required features into a computation and leaving the irrelevant ones outside the computation. Such separate representations likely lead into separate processing pathways as features are abstracted. Human vision has the characteristic of performing differently depending on the feature complexity of stimuli, as has been shown many times since Duncan and Humphreys (1989). Their experiments showed that in visual search tasks, difficulty increases with increased similarity of targets (that is, feature overlap and thus the ability to remove irrelevant features from the computation) to non-targets and decreased similarity between non-targets, producing a continuum of search efficiency. This is yet another form of a restrictive attentive process, that may be termed priming in this instance.

These^16^ achieve our goal, that is, to reduce the exponential complexity function to a much lower complexity expression, *O(2^M′^ P*′^3.5^). It is important to note that attentional selection to either select a single candidate or to restrict consideration to a small set of candidates forces a serialization of the problem solution. If the chosen candidate is correct, the algorithm of course terminates. However, if it is not, the next candidate must be selected for consideration. A related situation arises for stimuli that are not spatially localized (such as the examples of a star constellation or flock of birds given earlier) and in such cases, full image comparisons or more complex methods (such as piecing together results from the available sub-image matches) would be required, again perhaps necessitating a serial search. No single solution will handle all problem instances; different strategies can be applied in succession until success is achieved, each with a successively higher processing cost. This characteristic is unavoidable and representations must support the process.

This leads to the final stage of complexity level analysis, which is to determine what impact arises from the previous stages that provide the foundations for developing a theory of human vision. This impact is summarized here:

- Pyramidal abstraction affects the problem through the loss of location information and signal combination. It affects the problem solution by sometimes enabling shorter search processes, commonly known as coarse-to-fine search.
- Spatiotemporally localized receptive fields force the system to look at features across a receptive field instead of finer grain combinations and thus arbitrary combinations of locations must be handled by some other strategy.
- Attentional processes permit selection and restriction within the input data to control the overall size of input to be considered.

**Figure 2:**
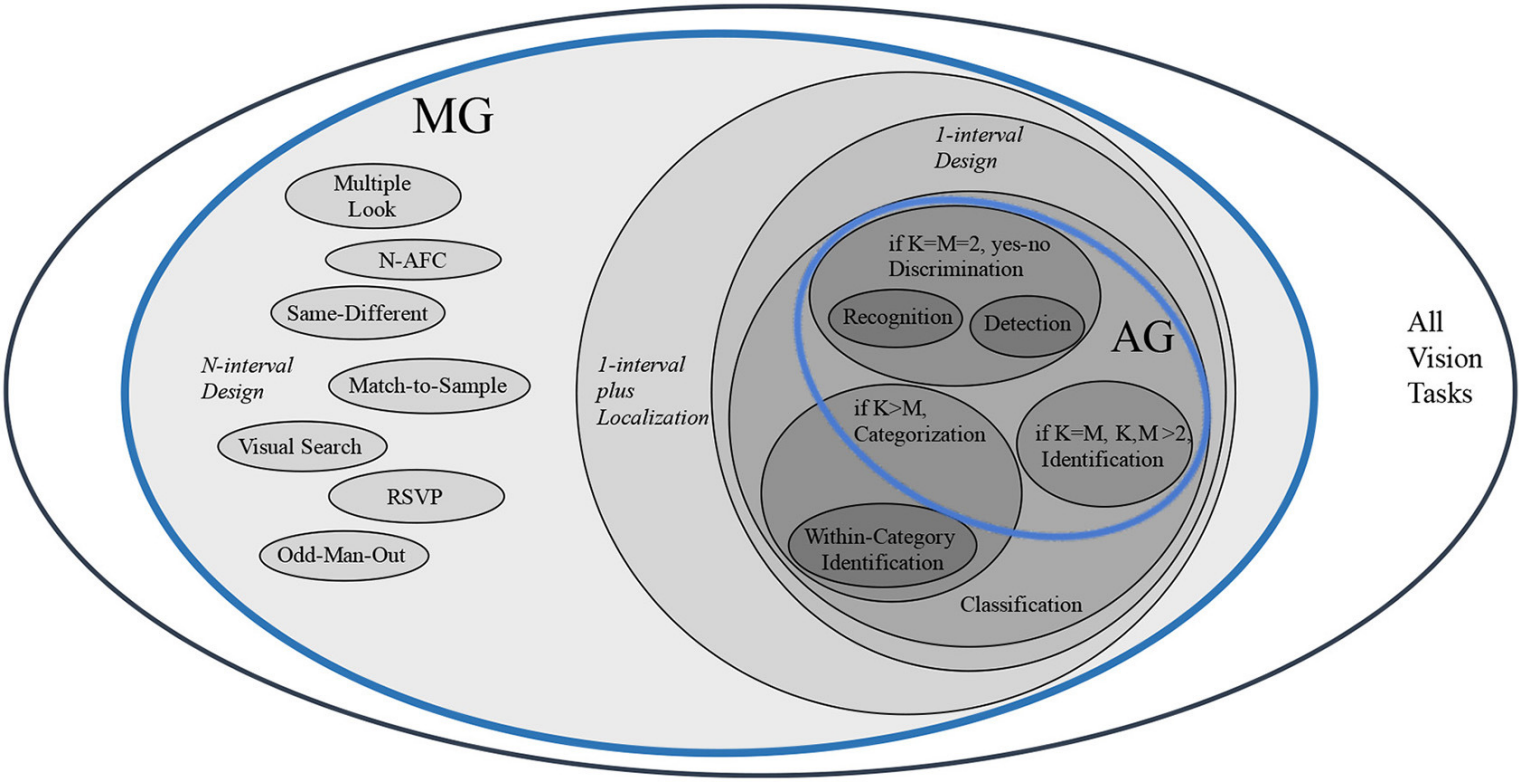
A taxonomy of visual tasks (adapted from Tsotsos, 2011 and task naming based on Macmillan and Creelman, 2005). Within each taxonomy element, there are both easy and difficult instances. AG, At-a-Glance tasks are those that can be solved using only a single feed-forward pass through the brain's visual processing machinery; MG, More-than-a-Glance tasks are those that require more processing than a single feed-forward pass through the brain's visual processing machinery; K, the number of possible images; *M*, the number of object categories of interest.

What this demonstrates is that although the analysis began considering solutions for the full space of problem instances, the need to fit a solution within the brain's resources forced a shrinking of that full space into something smaller. In other words, the restriction that Marr placed on his approach—that is, a clear figure-ground boundary—manifests itself as a restriction on the set of problem instances. Unfortunately, it is not easy to characterize this subspace. However, there is a possible taxonomy of visual tasks that can help. Figure 2 shows this taxonomy; there is no claim that it is complete. What it does point out is that the visual task most current AI systems address (such as Fukushima, 1988; LeCun and Bengio, 1995; Riesenhuber and Poggio, 1999; Krizhevsky et al., 2012), namely categorization, comprises only a small part of the taxonomy. It must be stressed that this taxonomy of tasks is not the same as a depiction of the space of problem instances. Each task has its own set of possible instances (and there may be overlap). For example, within categorization, there are instances that are easy (clear figure-ground boundary is seen) and instances that are difficult (without a clear figure-ground delineation).

To this point, the possibility of task influence on how a vision problem might be approached has not been discussed. The reason is that in his formulation, Marr discounted its use entirely and our approach was originally motivated by his perspective. However, increasingly, cognitive psychology and neuroscience has demonstrated that task influence plays a major role (see Carrasco, 2011; Tsotsos, 2011; Herzog and Clarke, 2014). In fact, accompanying the intractability proof in Tsotsos (1989) was a second theorem that showed that simple task knowledge can *bound* the search; it provides limits on the search space making it linear, rather than exponential, in the number of image elements (Wolfe, 1998 provides a relevant visual search review). The task knowledge can be as specific as target size or as generic as statistical regularities (as Parodi et al., 1998, illustrate empirically). This is a form of attentional priming (in advance of task execution) which limits what is processed in the location, feature and object domains. In Figure 2, task knowledge is critical for all the MG tasks as part of their basic definition, but also for the AG tasks since it bounds any search processes that might be employed in their solution. In effect, therefore, the original problem of Visual Match has been significantly reframed into a set of more specific problems as Figure 2 shows, with different constraints on the solution for each and together extending the temporal range of visual tasks far beyond Marr's 160 ms. This is consistent with van Rooij et al. (2012) who proposed computational-level theory revision as a way of dealing with intractability.

Thus, in addition to the three bullet points presented above regarding impact of the analysis, we add two more:

- The use of task or world knowledge can have profound impact on the computational complexity of a visual problem and should be employed whenever available (of course, there must be a default processing state when none is available),
- The discussion on different decision-making strategies and the complex taxonomy of visual tasks of Figure 2 strongly motivates the need for an executive control process that would dynamically decide on how to best approach and solve visual tasks as they are presented.

## The problems with pyramids

**Figure 3:**
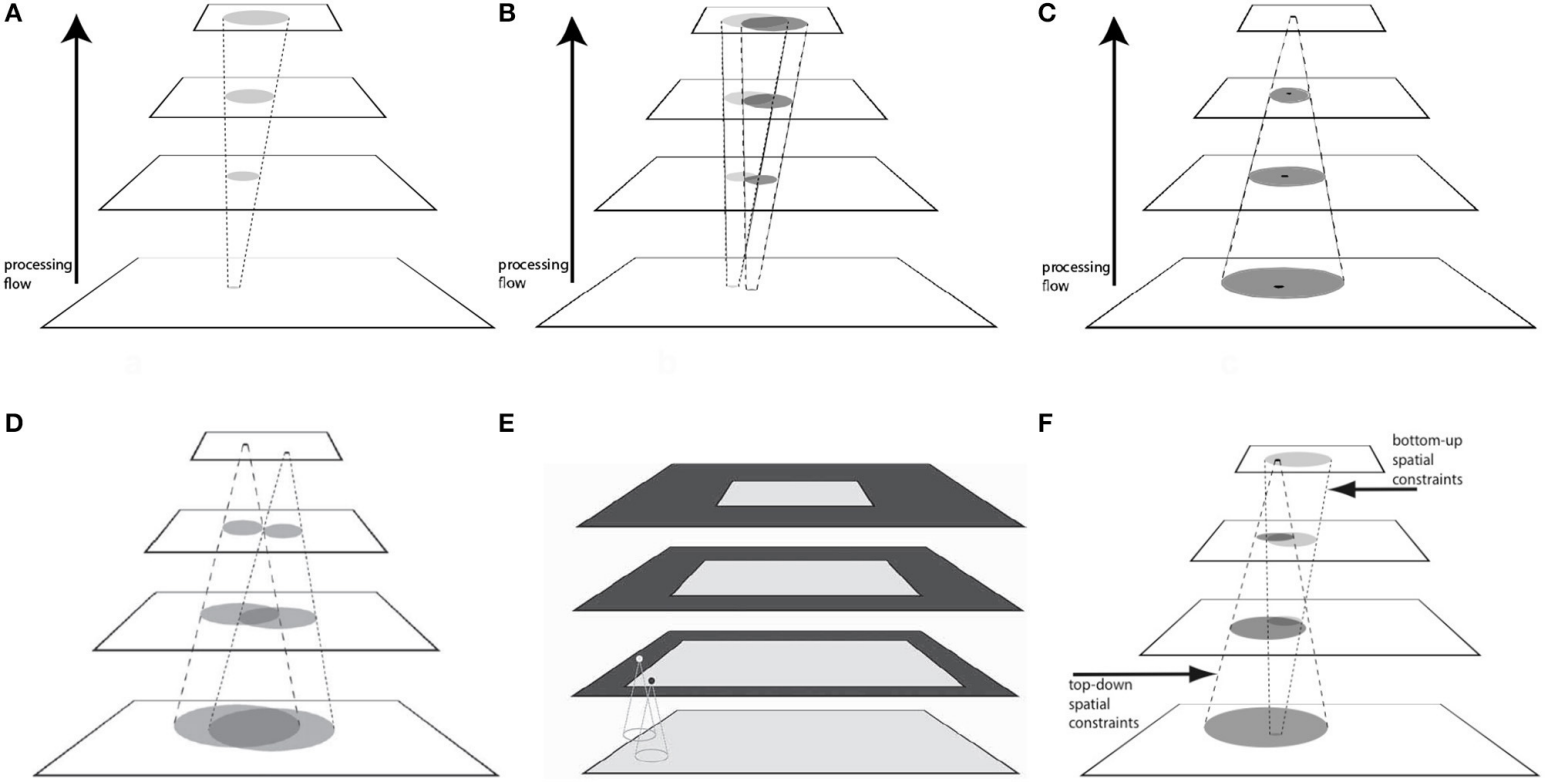
The breadth of problems inherent in pyramid representations. **(A)** The Blurring Problem. An input element in the lowest layer will affect, via its feed-forward connections, a diverging pattern of locations in the higher layers of the pyramid. **(B)** The Crosstalk Problem. Two input stimuli activate feed-forward projections that overlap, with the regions of overlap containing neurons that are affected by both. Those might exhibit unexpected responses with respect to their tuning profiles. **(C)** The Context Problem. A stimulus (black dot) within the receptive field of a top layer neuron, showing its spatial context defined by that receptive field. **(D)**. The Multiple Foci Problem. Regions of overlap show the extent of interference if two (or more) output nodes are considered simultaneously. **(E)** The Boundary Problem. The two units depicted in the second layer from the bottom illustrate how the extent of the black unit's receptive field is entirely within the input layer while only half of the receptive field of the gray unit is within the input layer. The bottom layer represents the retina; the next layer of the pyramid (say area V1) represents the spatial dimension of the viewing field in a manner that gives more cortical area to central regions than peripheral. The boundary problem forces more and more of the periphery to be unrepresented in higher layers of the pyramid. **(F)** The Routing Problem. Interacting top-down and bottom-up spatial search constraints are shown with the areas of overlap representing the viable search regions for best neural pathway. (Reproduced from Tsotsos, 2011).

Although pyramids played a strong role in reducing complexity, they do cause new problems with how information might flow within them. Some were first described in Tsotsos et al. (1995). Table 1 provides a characterization of each (more details can be found in Tsotsos, 2011) and the reader is encouraged to refer to Figure 3 while reading the table entries. These are all consequences of the basic connectivity pattern of Figure 1.

**Table 1:** A summary description of the main information flow problems resulting from pyramid representations.

**Problem**	**Data flow**	**Basic characteristic**
Blurring Figure 3A	↑	Feedforward neural connections have a diverging pattern, a one-to-many mapping, so that spatial precision is not preserved.
Crosstalk Figure 3B	↑	Two spatially separated stimuli each root a feedforward diverging cone of connections which may intersect thus presenting neurons within the intersection with a conflicted (corrupted with respect to the stimulus of interest) signal.
Context Figure 3C	↑	The receptive field of a neuron—a many-to-one mapping—in the higher layers of the pyramid can be potentially large enough to include not only a stimulus of interest but a significant local spatial context which may confound the stimulus interpretation.
Multiple foci Figure 3D	↑↓	If more than one neuron at the output layer is considered, the ability to tease their meanings apart depends on the spatial separation of the receptive fields (the inverted version of the crosstalk problem). In the forward flow direction, contexts due to each overlap to some degree, thus neural responses at the top cannot be considered independent. In the top-down direction, there is a complication when solving the routing problem (see part 3F) which although seemingly trivial for this simple example, would be quite difficult for scenes with many stimuli, such as natural scenes.
Boundary Figure 3E	↑	In a hierarchy of spatial convolutions, at each layer, a kernel half-width at the edge of the visual field is left unprocessed because the kernel does not have full data for its convolution. This is compounded layer by layer because the half-widths are additive layer to layer. The result is that a sizeable boundary region at the top layer is left undefined (a true information loss) and thus the number of locations that represent veridical results of neural selectivity from the preceding layer is smaller and restricted to the central portion of the visual field. Solutions, such as used in current CNN's were first described in van der Wal and Burt (1992); they have no biological counterpart. See Tsotsos (2011) and Tsotsos et al. (1995, 2016) for a theory on how the brain deals with the boundary problem.
Routing Figure 3F	↑↓	Because of the above problems, a difficulty arises in the search for the neural pathway that connects a stimulus to the neurons that best represent it. If the search is bottom-up—from stimulus to highest layer neuron—then the search is constrained to the feed-forward cone outlined by the dotted lines. If the decisions are based on locally maximal neural responses (such as max pooling), then there is nothing to prevent a bottom-up search losing its way, due to the diverging feedforward connectivity, and missing the globally maximum response at the top layer. It is clear that to be successful, the correct path must always go through the overlap regions shown in dark ovals. But nothing guarantees that the local maximum must lie within those overlap regions. If the search is top-down—from the globally maximum responding neuron to the stimulus—the search is constrained by the dashed lines. Only top-down search is guaranteed to correctly connect the best responding neuron at the top with its stimulus because the search is constrained by the connectivity pattern of the source neuron which necessarily contains the goal stimulus.

*Other such problems, not described here are the Sampling, Lateral Spread, Spatial Spread, Spatial Interpolation, and Convergent Recurrence problems and the interested reader can find these in Tsotsos (2011)*.

The consideration of representational issues, such as the problem with information flow in a pyramid is not common in the modeling literature (but see Anderson and Van Essen's Shifter circuits, 1987, that were strongly motivated by information routing issues). For the most part, the information flow problems require dynamic solutions that change from moment to moment depending on task and input. Models that ignore these routing characteristics are not only incomplete but lose out on incorporating the constraints that arise.

## Lattice of pyramids

The pyramid representation as described so far fits very naturally into the hierarchical view of Hubel and Wiesel (1965, 1968). However, it is insufficient. Felleman and Van Essen (1991) give a set of criteria for determining hierarchical relationships among the visual areas in the cortex. These are:

“each area must be placed above all areas from which it receives ascending connections and/or sends descending connections. Likewise, it must be placed below all areas from which it receives descending connections and/or sends ascending connections. Finally, if an area has lateral connections, these must be with other areas at the same hierarchical level.”

**Figure 4:**
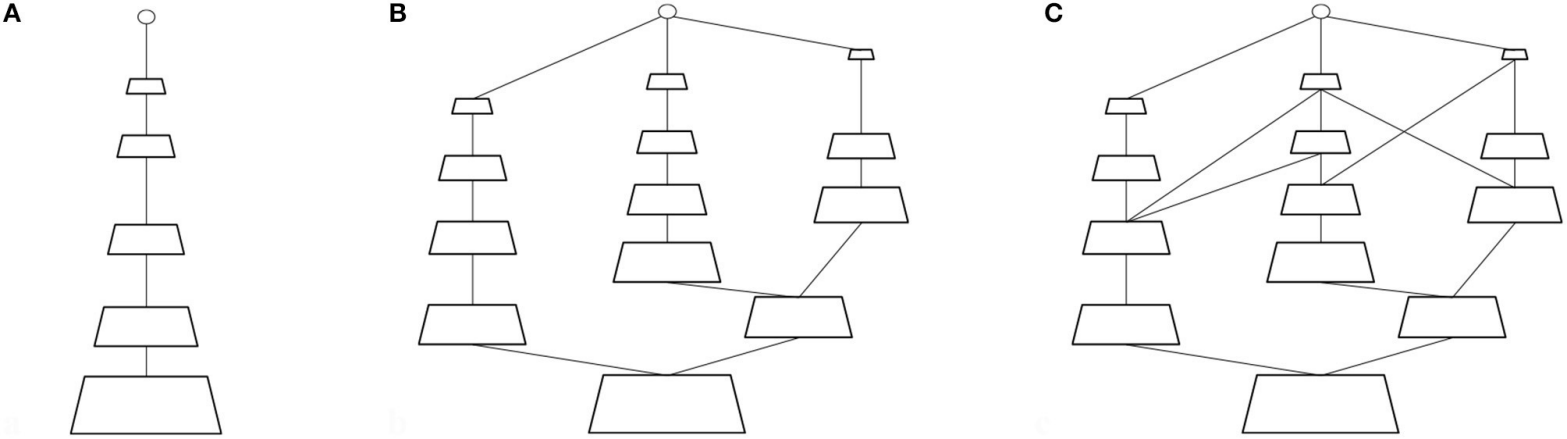
From pyramids to P-lattices. **(A)** A simple pyramid representation. **(B)** A lattice of three pyramids. **(C)** A lattice of pyramids showing complex connectivity.

This characterization of connectivity resembles that of a general lattice, as shown in Figure 4B (see Birkoff, 1967, for a mathematical discussion on the properties of lattice structures). In contrast to the pyramid of Figure 4A, i.e., exactly the representation found in convolutional neural networks (CNN-see LeCun and Bengio, 1995; Riesenhuber and Poggio, 1999; Krizhevsky et al., 2012), Figure 4B highlights the fact that there may be more than one pathway from input, as is well-documented in visual cortex. Tsotsos (2011) marries the concept of the pyramid with that of the lattice to define the P-Lattice, or lattice of pyramids in order to fully accommodate the criteria laid out by Felleman and Van Essen.

Each element or layer of the pyramid will be referred to as a *sheet*—an array of retinotopically organized neurons of common tuning profile. Each sheet may be connected to more than one other sheet in a feed-forward, recurrent or lateral manner. The main constraint is that no matter which path is taken from lower to higher level, each sheet at a lower level has the same or larger number of elements compared to any higher-level sheet on its path. Both Figures 4B,C are P-Lattices; the Figure 4C shows a more complex version of Figure 4B in order to illustrate the full nature of the representation. The formalization will not be further described, but is developed in Tsotsos (2011). It should be apparent that the P-Lattice representation is much more faithful to the organization of different processing areas in the brain than the standard CNN.

The P-Lattice concept also lends itself very naturally to thinking about an organization that includes not only a part-whole relationship as is common for pyramids, but also a specialization relationship. Different features may be separated out into different sheets, and those may then be specialized differently along each pathway of the P-Lattice.

## Selective tuning

As a result of the complexity level analysis, a series of papers outlined the development of a model for how the main conclusions in the previous sections might impact a visual processing hierarchy (Tsotsos, 1988b, 1990, 1995b, 2011; Tsotsos et al., 1995, 2001; Rothenstein and Tsotsos, 2014). This model, named Selective Tuning (ST) was intended to provide a mechanistic explanation for how not only attentive selection and restriction might occur, but also, how the visual system deals with the many problems of information flow described in the previous section. To this end, ST incorporated pyramid representations, spatiotemporally limited receptive fields, separable feature representations, dynamic tuning and attentive selection. In order to deal with the Context Problem, ST employs a suppressive mechanism, recurrent localization, to inhibit portions of a receptive field deemed ‘ground’ while attending to ‘figure’ (see Tsotsos et al., 1995; Tsotsos, 2011 for details). Thus, suppression must be added to selection and restriction to form the full suite of attentional mechanisms. ST also offers an explanation for a wide variety of attentional phenomena; it is among the oldest and most studied models of attention. ST, beginning with the earliest papers, made a number of predictions about visual attention at both neural and behavioral levels, which, starting in the late 1990's, have seen broad and strong experimental support^17^ (reviewed in Tsotsos, 2011; also in Hopf et al., 2010; Carrasco, 2011 and more).

**Figure 5:**
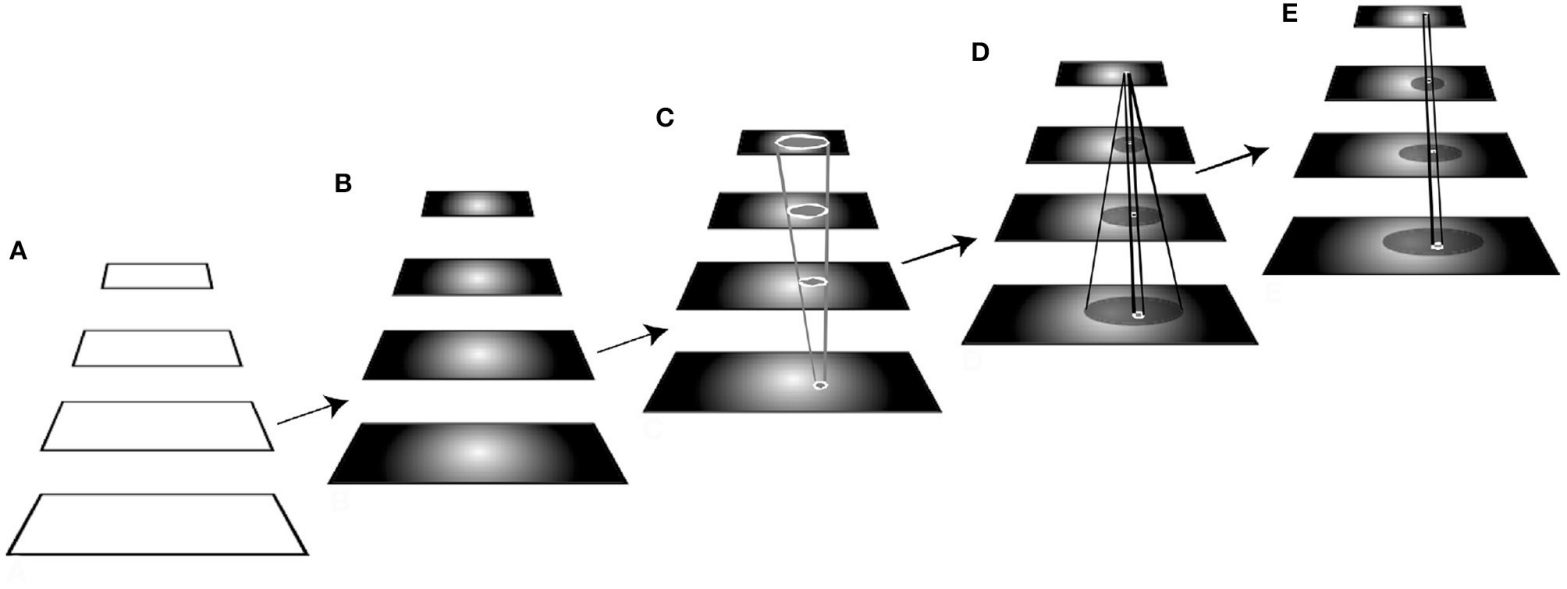
The different stages of processing of the visual hierarchy needed for different visual tasks. The five components of the figure represent processing stages ordered in time, from left to right. **(A)** In the first stage, the network is portrayed as “blank,” that is, without stimulus or top-down influences, as it might be prior to the start of an experiment, for example. **(B)** The second stage shows the network affected by a top-down pass tuning the network with any priming information to set up its expectation for a stimulus to appear, when such information is available. Here, the network is set up to expect a stimulus that is centrally located and is imposed via a global suppression of non-task-relevant locations and/or features. **(C)** At this point, the stimulus appears and is processed by the tuned network during a single feedforward pass. If the task is sufficiently simple, such as a detection or categorization tasks with sufficiently simple stimuli so that figure can be selected from ground, processing is complete. **(D)** If the required task for this stimulus cannot be satisfied by the first feedforward pass, such as for a within-category identification or the need for an eye movement response, the recurrent localization algorithm is deployed that traverses the network in a top-down manner, identifying the selected components while suppressing their spatial surrounds locally. **(E)** A subsequent feedforward pass then permits a re-analysis of the attended stimulus with interfering signals reduced or eliminated. It also permits a continuation of the cycle in a repeating fashion, such as would be needed for visual search. This illustrates the main cost associated with dynamic tuning, namely, time. Different visual tasks require different processing times depending on passes through the hierarchy. A smaller additional cost would be the process of actual tuning. (Reproduced from Tsotsos and Kruijne, 2014).

Figure 5 illustrates the main features of the model showing how there are many aspects to attentive processing, and which are executed determined by the nature of the task of the moment. It shows the different stages of processing of the visual hierarchy needed for different visual tasks. The five components of the figure represent processing stages ordered in time, from left to right. The stages may be described as Figure 5A: pre-stimulus (shown as blank to portray a visual hierarchy ready for a new stimulus); Figure 5B. top-down priming for task; Figure 5C: feedforward stimulus processing and figure selection; Figure 5D: recurrent localization and local suppression, if the task requires it; Figure 5E: secondary feedforward processing. This illustrates the main cost associated with dynamic tuning, namely, time. Each hierarchy traversal may be primed for different function. Different visual tasks require different processing times depending on passes through the hierarchy. A smaller additional cost would be the process of actual tuning. Different visual tasks require different sets of these basic elements, sometimes with repeated elements and this shows how *dynamic tuning* can be realized.

To summarize, ST features several major elements not present in other models of attention: (1) the recurrent localization process; (2) the integration of multiple attentional processes within a single framework; (3) both local and global attentional operations; (4) the realization that not all vision occurs within the 150 ms time frame and that different kinds of visual tasks require different processes and thus take different durations to complete; (5) the capacity to dynamically tune the visual processing hierarchy depending on task; and (6) the use of inhibitory mechanisms rather than enhancement in order to achieve attentive effects (enhancement is a side-effect of suppression of competing stimuli).

## Nature of signal interference in the P-Lattice

The impressive successes of deep learning approaches to vision system development may lead one to think that vision is a solved problem, and that all one needs is a fast-enough computer and enough training data^18^. The complexity level analysis does indeed tell us something of interest here: that with enough computational capacity, *some* vision problems can be solved. Recall that the role of image size in the complexity function; this dictates the primary barrier without attentive selection. Proponents of deep learning widely acknowledge that the advent of GPU's and faster processors contributed to the recent successes. This is not the same as saying the vision problem has been made tractable: all it means is that with enough GPU power, the size of image—that is, the value of *P* that can be realized in the complexity expression—is now a reasonable number for practical applications. Importantly, it cannot be as large as the size of a human retina. We also note that although those approaches do indeed receive some motivation from biological vision, that motivation is almost entirely based on knowledge of the late 1960's. The methods validate the concepts of spatially limited receptive field size, convolution processing and hierarchical processing levels, but not much more. The representations typically used in deep learning are also not easily related to neural representations nor their methods for decoding those representations. None of this of course should detract from their practical success. The point here is simply that there is a great deal more work to be done with respect to understanding how biological systems deal with visual problems.

Let us return to the representation problem. Pyramid representations help with reducing complexity but as shown above, add new complications that can, as a group, be considered as signal interference. In other words, all incoming signals are represented in all layers of a pyramid (this is true for central regions, but not for peripheral—see Figure 2E), as they are in all layers of a modern CNN too. But they are not easily discriminable due to the interference that the context, boundary, blurring problems impose. It is important to examine interference more deeply.

The Context Problem is due to many-to-one neural mapping, the Blurring Problem due to one-to-many neural mapping and the Boundary Problem due to the realities of convolution processes. Of these, only the Boundary Problem leads to actual information loss and specifically in the periphery; the rest lead to signal interference via combination. Every signal continues to be represented during the feedforward traversal of an input signal, except that it becomes increasingly intertwined and amalgamated with nearby signals, dictated by receptive field sizes. Modern theories prescribe computational decoding procedures that are able to take this muddled representation as input and decode it to extract meaning. For example, Hung et al. (2005) used a classifier-based readout technique (linear SVM) to interpret the neural coding of selectivity and invariance at the IT population level. The activity of small neuronal populations over very short time intervals (as small as 12.5 ms) contained accurate and robust information about both object “identity” and “category.” Coarse information about position and scale could be read out over three positions. Isik et al. (2014) used neural decoding analysis (also known as multivariate pattern analysis, or readout) to understand the timing of invariant object recognition in humans. Neural decoding analysis applies a machine learning classifier to assess what information about the input stimulus is present in the recorded neural data. They found that size—and position-invariant visual information appear around 125 and 150 ms, respectively, and both develop in stages, with invariance to smaller transformations arising before invariance to larger transformations. They claimed that this supports a feed-forward hierarchical model of invariant object recognition where invariance increases at each successive visual area along the ventral stream. This is in contrast to work by Zhang et al. (2011) who show how a classifier can be trained on data from isolated-object trials and then make predictions about which objects were shown on either different isolated-object trials or on trials in which three objects are shown. They concluded that by focusing on how information is represented by populations of neurons, competitive effects that occur when two stimuli are presented within a neuron's RF, and global gain-like effects that occur when a single stimulus is presented within a neuron's RF, can both be viewed as restoring patterns of neural activity for object identity and position information, respectively. The competitive interactions Zhang et al. refer to are attentive mechanisms whose intent is to reduce interference, which was the goal of their study. The difference between the last two papers is due to the different stimuli used, the latter requiring attention and the former not. We can conclude that although coarse location information is likely easily extracted after a single feedforward pass for detection tasks, more complex visual tasks that require image details of precise features of location likely are not. The Multiple Foci problem of Figure 3D illustrates this nicely; spacing within the visual field dictates the degree of interference.

**Figure 6:**
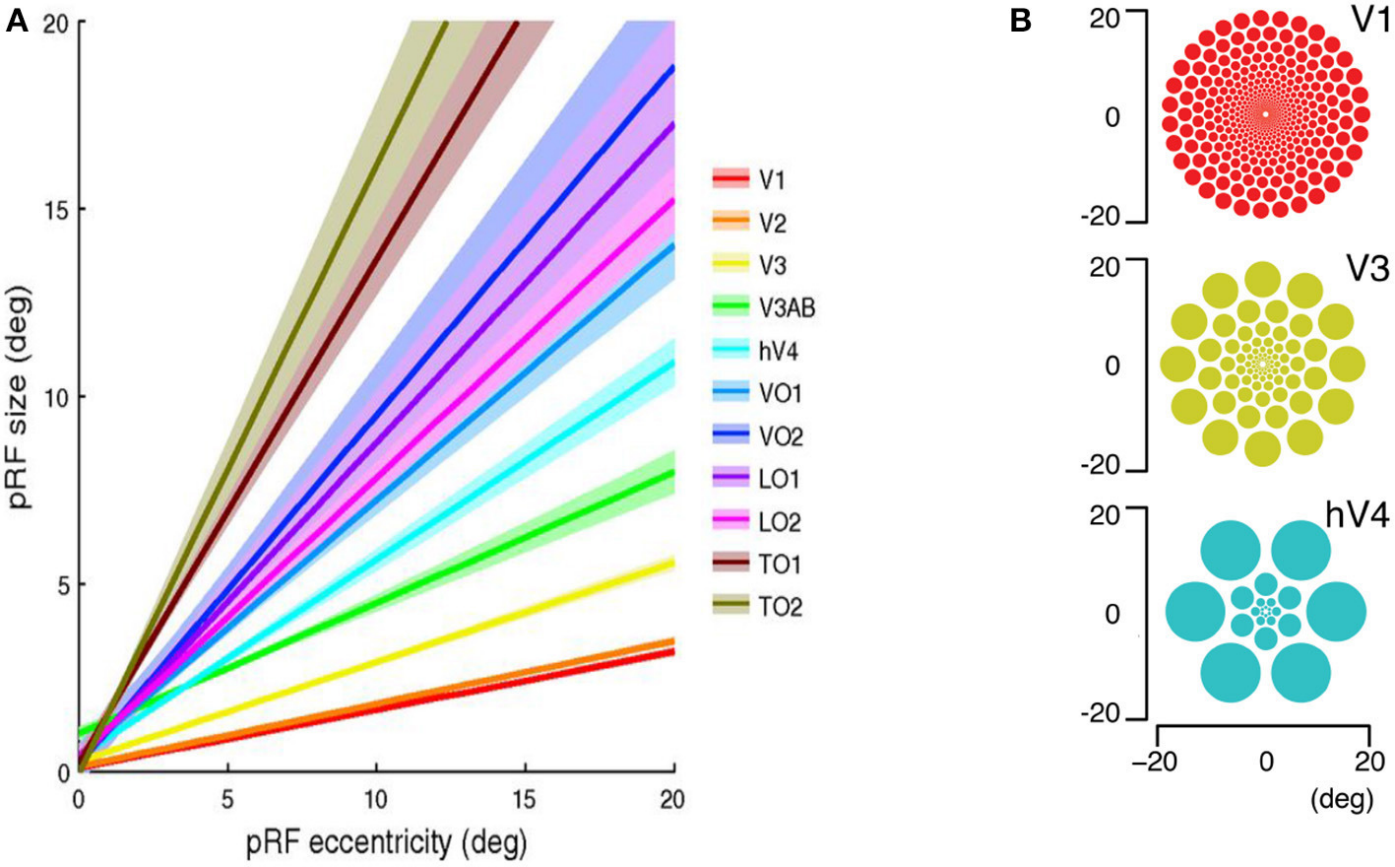
Regularities in human population receptive field properties measured with functional MRI. **(A)** Population receptive field size as a function of eccentricity in several human retinotopic maps. Two clear trends are evident. First, the population receptive field size increases with eccentricity within each map. Second, the population receptive field size differs between maps, with the smallest pRFs in V1, and much larger pRFs in ventral (hV4, VO-1/2) and lateral (LO-1/2, TO-1/2) maps **(B)** The spatial array of pRFs using the parameters in the left panel. The radius of each circle is the apparent receptive field size at the appropriate eccentricity. [a-from Kay et al., 2013, Reproduced with permission of the publisher; b-Reproduced with permission of J. Winawer (https://archive.nyu.edu/handle/2451/33887)].

**Figure 7:**
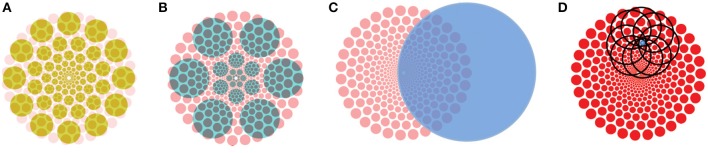
Superposed spatial arrays of receptive fields using plots of Figure 6. **(A)** V3 over V1. **(B)** V4 over V1. **(C)** LO1 over V1 with the LO1 receptive field centerd over 20° eccentricity to match the data from Figure 6. **(D)** The black circles represent the feedforward divergence of outputs from a single V1 neuron at the V4 level.

Let's continue to examine this neural interference. It is well-known and studied that the size of visual receptive fields generally increases with higher levels (or greater abstraction) of processing within the visual hierarchy of the brain. There is a further dependency not only on abstraction level but also eccentricity, or distance of the receptive field from the center of gaze. Kay et al. (2013) provide illuminating plots of receptive field sizes in many visual areas of human cortex as a function of retinal eccentricity, reproduced in Figure 6. It is clear that the receptive field size increases with eccentricity within each visual map. Second, the receptive field size differs between maps, with the smallest pRFs in V1, and much larger pRFs in ventral (hV4, VO-1/2) and lateral (LO-1/2, TO-1/2) maps, showing a progression from least to most abstract in terms of processing. It is important to note—as the complexity level analysis pointed out earlier—that receptive fields are space-limited, i.e., there seem to be no fully connected layers where all receptive fields are connected to all others. There is a well-defined feedforward as well as feedback connectivity pattern (mostly symmetric) so that each element of a representation affects a clear feedforward diverging cone of elements in the next representation, is fed by a clear converging cone of elements from the earlier representation and these connections are bidirectional (this is exactly what Figure 1 illustrates). A re-plotting of the elements of Figure 6 leads to an explicit view in Figure 7 of the spatial extent of feedforward convergence. Superimposing the receptive field maps, V3 onto V1, V4 onto V1 and a hypothetical LO1 receptive field (using values from Figure 6 at 20° eccentricity) shows clearly that degree of signal convergence onto single neurons with higher levels of visual processing in cortex. These figures are a concrete demonstration of the Blurring and Context Problems of Figure 3. How can the visual system function at all under such circumstances? Most models do not consider how such eccentricity-dependent receptive field size variations might be usefully incorporated.

First, it might be the case that there are many more target representations at higher levels than previously thought, something hinted at by the very recent results of Glasser et al. (2016). That is, the breadth of the P-Lattice representation in the brain may be significant. Perhaps these might be specializations as suggested earlier, thus removing some of the interference that way. Second, lateral interactions within representations could assist in well-known ways by enhancing contrast, contrast in this case not being restricted to luminance but to contrast in any featural or conceptual space. But this contrast enhancement cannot be total because local decisions may be wrong (Marr's, 1982 principle of least commitment; Herzog and Clarke, 2014).

It is not hard to believe that a classifier can indeed be trained to extract location for simple (Marr-like) images with small numbers of separated stimuli as Hung et al. report. But such a situation is not representative of real vision. Something more is needed for natural images and for tasks where more precision is required than simple coarse position. There are really two choices: 1-provide mechanisms that dynamically ameliorate the interference before interpretation; or, 2-provide mechanisms to correctly interpret corrupted representations. The methods just described are of the latter type. We chose to explore the former possibility. A key feature of the Selective Tuning model of visual attention is the use of a recurrent localization process that imposes a suppressive surround around the attended stimulus as shown in Figure 5D (Carrasco, 2011; Tsotsos, 2011) to deal with the Context and Routing problems. This would require a top-down pass through the processing hierarchy after the initial feedforward pass, consistent with the behavioral timing observed for such tasks. The requirement for an additional top-down pass for localization is not inconsistent with the claims of Isik et al. (2014). In ST, it is the recurrent localization process that replaces the role of the classifier, and in contrast to current classifiers presents a biologically plausible mechanism (supported experimentally, e.g., Boehler et al., 2009, 2011; Hopf et al., 2010).

Signal interference within a pyramid representation is a reality that seems insufficiently addressed in general. To be sure, the majority of experimental work, whether neural or behavioral, focus on foveal or near-foveal stimuli and as the plots of Figure 6 show, the interference impact is not so great. Further, most experimenters use relatively simple stimuli, spaced apart and with little conflicting context. As the diagrams of Figure 3 show, the distance between stimuli matters for the Blurring, Crosstalk, and Context Problems and it is experimentally possible to minimize the effect, thus making it appear as if the problem does not exist. As a result, experimental work does not fully address the problem in order to determine if and how it might cause interference or how the brain might deal with it. New experimental paradigms seem required.

## Attentive processing and adaptive beamforming

**Figure 8:**
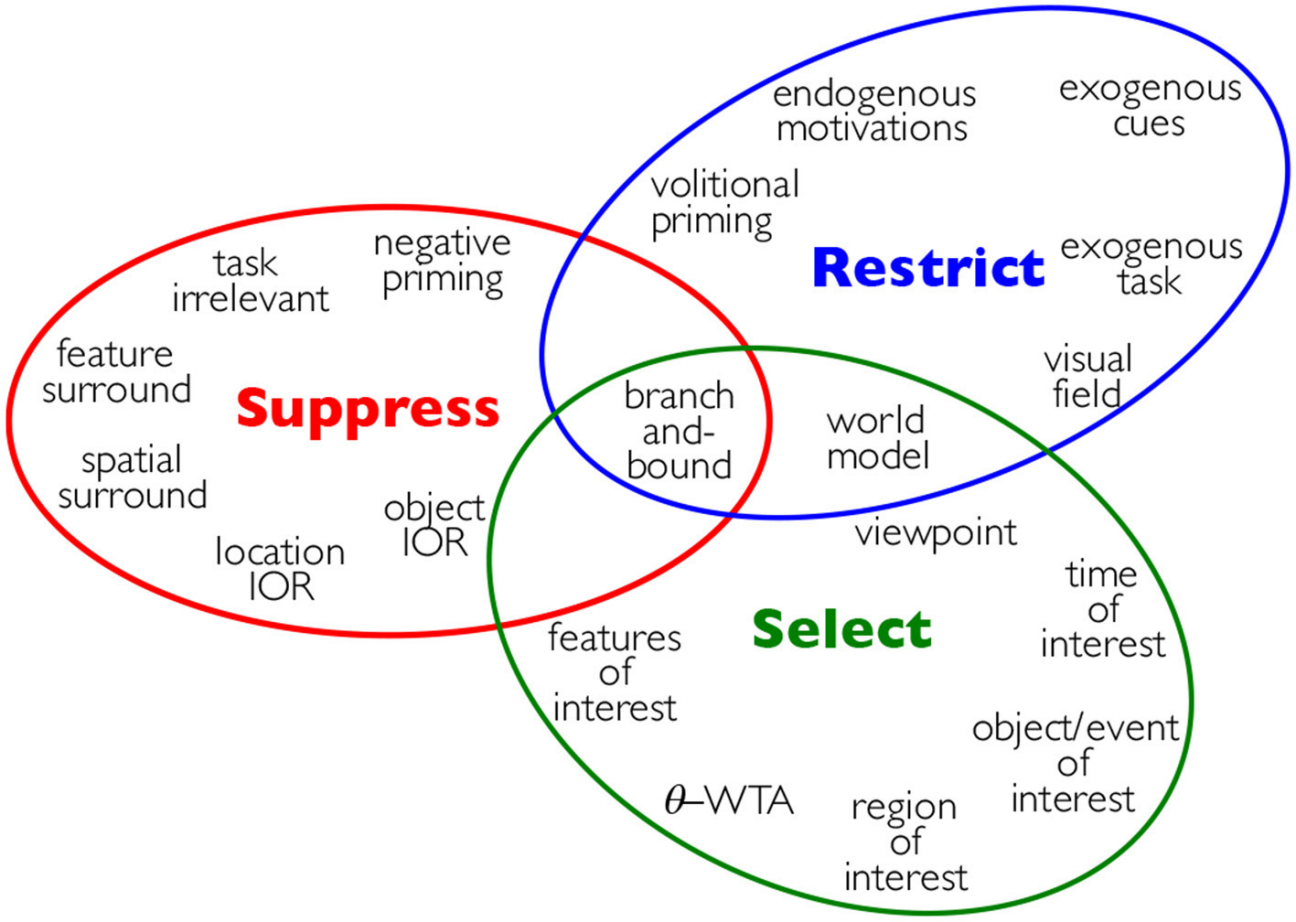
Attention is a set of mechanisms that tune and control the search processes inherent in perception and cognition, with the major types of mechanisms being Selection, Suppression, and Restriction. See Tsotsos (2011) for details on each of the sub-mechanisms.

The most common way in which attention has found its way into theories and models of visual processing or other human sensory or cognitive abilities is as a mechanism to defeat capacity limits. This is also true for computational systems. The most prevalent mechanism is that of selecting a region of interest in some modality of the sensory input or in some conceptual space, such as a task-relevant sub-domain of interest. In a behaving agent, eye movements are most often considered the primary indicator of a shift in attention. Nevertheless, as Tsotsos (2011) argues and as any review of visual attention (such as Carrasco, 2011) amply illustrates, attention is a much broader capability with, sadly, no real consensus on how it might be characterized. One possibility for such a broad characterization appeared in Tsotsos (2011) where is was proposed that attention is a set of mechanisms that tune and control the search processes inherent in perception and cognition, with the major types of mechanisms being Selection, Suppression, and Restriction. Within each type are several specific mechanisms as shown in Figure 8.

Earlier, as a result of the complexity level analysis, it was asserted that the original vision problem is reframed by partitioning the space of problem instances into sub-spaces where each might be solvable by a different method instead of having a single, optimal, algorithm for all problem instances. The resource limits—which are fixed and common for all sub-problems in the case of the brain—guide the choices. A key element of the process is to have a method that, when confronted with a visual problem instance, can quickly determine which solution method to apply. And this is where attention is critical. A sufficiently flexible attentive process can start from the general and thus largest possible problem definition, and then focus in and scale down the problem to more manageable sub-problems. Combining all of these seemingly disparate tools, as shown in Figure 8, within a single formulation seems a daunting task, but this is what the Selective Tuning model of attention attempts to do (Tsotsos, 1988b, 1990, 2011; Tsotsos et al., 1995).

Interestingly, a related combination of disparate tools has not only been attempted previously, but has developed into a well-understood and very widely use technology, namely adaptive beamforming. Beamforming is a signal processing technique used in sensor arrays for directional signal transmission or reception (Van Veen and Buckley, 1988). Electromagnetic waves are additive and if more than one wave co-exists in space and time, this additive property causes each waveform to interfere with the others. Beamforming attempts to minimize this interference. This is achieved by controlling how elements combine so that some signals experience constructive interference while others experience destructive interference. Beamforming can be used at both the transmitting and receiving ends in order to achieve spatial selectivity. Beamforming can be used for radio or sound waves and has found numerous applications in radar, sonar, seismology, wireless communications, radio astronomy, acoustics, and biomedicine. An adaptive beamformer dynamically adjusts in order to maximize or minimize a desired parameter, such as signal-to-interference-plus-noise ratio. Dynamically adjusting phase and magnitude will cause the antenna gain pattern to change and provides for directional sensitivity without physically moving an array of receivers or transmitters.

The essence of beamforming seems precisely what attention seeks to accomplish: to pick out the relevant signal from among all the irrelevant ones. This connection between attention and beamforming has been made previously in the auditory domain (see Kidd et al., 2015, for a recent effort) in order to provide solutions to the well-known Cocktail Party problem. There are components of constructive and destructive interference within the attentional mechanisms of ST, and more, but it would be beyond the scope here to further explore the relationship. However, it is clear that any representations of visual information processing must support these mechanisms. Adaptive beamforming—or perhaps more appropriately *attentive beamforming*—might present an appropriate analogy for formalization of dynamic visual attention processes.

## Conclusions

The hallmark of human vision is its generality. The same brain and same visual system allow one to play tennis, drive a car, perform surgery, view photo albums, read a book, gaze into your loved one's eyes, go online shopping, solve 1,000-piece jigsaw puzzles, find your lost keys, chase after your young daughter when she appears in danger, and so much more. The reality is that incredible as the AI successes so far have been, it is humbling to acknowledge how far there is still to go. Recent AI systems even sometimes outperform humans so it is difficult to determine how well they might provide an explanation for human intelligence. With respect to an explanation for human intelligence, it is as important to ensure that model systems behave correctly as humans and with the same response times, as it is to ensure model systems fail as humans do. The successes have all been uni-taskers (they have a single, narrowly defined function)—the human visual system is a multi-tasker, and the tasks one can teach that system seem unbounded. And it is an infeasible solution to simply create a brain that includes a large set of uni-taskers.

Representation has been central to AI since its inception and it is only recently that it seems supplanted by the success of the machine learning approach. Unfortunately, the representations that learning systems create—except possibly for limited aspects of early vision—seem inscrutable. It might be that in order to make progress, there remains a need to better understand the kinds of representations and their transformations as they may be occurring in the brain, a sentiment appearing decades ago. Zucker (1981) stressed the importance of representation. He pointed out that computational models have two essential components—representational languages for describing information, and mechanisms that manipulate those representations, and: “*One of the strongest arguments for having explicit abstract representations is the fact that they provide explanatory terms for otherwise difficult (if not impossible) notions.”*

Our presentation has focused on the constraints that complexity level analysis presents for the representations and for the visual processes that operate on them in the brain (or in machines). It is clear that the main claim, namely, that resource-complexity matching is a source of critical constraints on the viability of theories, remains intact. The 30 years that have passed since their first introduction in this context have given us the luxury of seeing how they stood the test of time. None of the conclusions were in common use back then and some indeed were firmly believed to be incorrect^19^. Throughout, we have argued for a very specific view on representation and their processing, whose features include:

- an overall organization of visual areas into a lattice of pyramids,
- spatiotemporally limited receptive fields,
- specialized pathways based on visual features,
- a suite of attentional mechanisms that dynamically suppress, select and restrict processing to control the input space and to ameliorate the signal interference problem, and,
- the use of task or world knowledge can have profound impact on the computational complexity of a visual problem and should be employed whenever available,
- a partitioning of the space of visual tasks into a taxonomy of sub-tasks, each with its own specific characteristics and requiring differing methods all realized on that same processing substrate,
- the different decision-making strategies and the complex taxonomy of visual tasks strongly motivates the need for an executive control process that would dynamically decide on how to best approach and solve visual tasks as they are presented.

Moreover, the intractability results of our own work and of all other authors cited here, and more, show the futility of pursuing single criterion algorithms of any kind (for example, Friston's (2010) free-energy principle). Much is already in line with current knowledge of the brain, many of these features have found their way into the successful systems of the present, but much still requires further study. There is no suggestion that complexity level analysis can replace any other type of analysis. However, it is a critical component of theory development and provides an important source of constraint that models cannot do without.

## Author contributions

The author confirms being the sole contributor of this work and approved it for publication.

### Conflict of interest statement

The author declares that the research was conducted in the absence of any commercial or financial relationships that could be construed as a potential conflict of interest.

## Footnotes

^1^There is a distinct focus on our own work throughout this paper simply because the goal of this presentation is to examine that old work and how its conclusions have stood the test of time. This is not to say that no other work has appeared since nor that all other work is unimportant. Far from it! However, most other developments along complexity theoretic lines do not line up with the main thread of this paper, namely, what can this analysis tell us about representations in the brain.

^2^Details on this assertion are beyond the scope of this paper. The interested reader can find a very accessible discussion in Stockmeyer and Chandra (1988), while those wishing a deeper treatment should see classic texts such as Garey and Johnson (1979), Papadimitriou (2003).

^3^Decidability is discussed in Davis (1958, 1965). Proof of *decidability* is sufficient to guarantee that a problem can be modeled computationally. It requires that the problem be formulated as a decision problem and that a Turing Machine is defined to provide solution. This formulation for the full generality of vision does not currently exist. If no sub-problem of vision can be found to be decidable, then it might be that perception as a whole is undecidable and thus cannot be computationally modeled. However, many decidable vision problems are mentioned throughout this paper so that is not the case.

^4^It is not within the scope of this paper to detail the full sequence of papers on the topic, so they are simply cited here so that the interested reader can examine them separately: Tsotsos (1987, 1988a,b, 1989, 1990, 1991, 1992, 1993, 1995a, 2011), Ye and Tsotsos (1996), Ye and Tsotsos (2001), Parodi et al. (1998) Andreopoulos and Tsotsos (2013).

^5^A *problem* is distinct from an *algorithm*. A problem is a general statement about something to be solved (Marr's computational level, Marr, 1982) whereas an algorithm is a proposed solution (Marr's representational and algorithmic level). One can address computational complexity at both levels: the inherent difficulty of a problem in its general form as well as the difficulty of a particular algorithm. Problem complexity applies to all possible solutions and any realization of them while algorithm complexity applies only to the specific algorithm analyzed. Here, we address only the former.

^6^If we look at the perceptual task definitions provided by Macmillan and Creelman (2005), we see that all psychophysical judgments are of one stimulus relative to another — the basic process is comparison. The most basic task is termed discrimination, the ability to tell two stimuli apart. The fact that it is a sub-element of all visual tasks means that the difficulty of any visual task is at least as great as that of this sub-element. Interestingly, this is a decidable perceptual problem and is an instance of the Comparing Turing Machine (Yasuhara, 1971). Further discussion is found in Tsotsos (2011).

^7^Although it is admittedly unusal to include this restriction, it makes sense if one wishes to follow the Marr approach to vision, i.e., that visual processing included no top-down or knowledge-based guidance. Marr (1982; p 96) restricted his approach to be applicable for the first 160ms of processing by the brain and for stimuli where target and background have a clear psychophysical boundary. Our original motivation was to show that this approach would not suffice for all stimuli; this was successfully accomplished.

^8^In general, it is true that for problems that are proven to have such complexity characteristics, it only means that sufficiently large problem instances may not be realizable and that perhaps small ones, or particular subsets or special cases of the overall problem, may be perfectly realizable. The point of the complexity proof is to characterize a general solution that applies for all possible instances. For vision, this is a tall order. The space of all possible images is impossibly large. Pavlidis (2014) derives possible characterizations of this space. He claims that a very conservative lower bound to the number of all possible human-discernible images is 10^25^ and may be as large as 10^400^. The practical import is that any solution that one proposes must apply to this full set.

^9^Kube (1991) pointed out that the Knapsack problem, which forms the foundation of the proof, is known to have efficient solutions under certain circumstances. Tsotsos (1991) surveys those efficient solutions and notes that they are not easily matched to, let alone relevant for, biologically plausible architectures and processes. It is beyond the scope to give further details on this here but the sequence of commentaries in Tsotsos (1990, 1991), Kube (1991) provide more detail.

^10^Traub (1991) also struggles with this issue. He suggests that a theory of complexity of scientific problems is needed such that formulations capture the essence of the science and that they be tractable.

^11^This is essentially the same process as seen in Judd (1988), van Rooij et al. (2012), van Rooij and Wareham (2012), and others, where they effectively used intractability results to guide a search for methods and problem re-formulations that would lead to realizable solutions. However, a major difference is the need to further constrain that search to be consistent with neuroanatomical and neurophysiological knowledge.

^12^The notation *O(-)*, known as Big-O notation, signifies the order of the time complexity function, that is, its dominating terms asymptotically.

^13^Garey and Johnson (1979) detail a variety of strategies and heuristics for dealing with intractable problems theoretically and these are as applicable here as for theoretical computer science problems.

^14^One additional possibility is that of a fixed parameter-tractable algorithm, that is, an algorithm that is exponential only in the size of a fixed parameter while polynomial in the size of the input (see Downey and Fellows, 1999; van Rooij and Wareham, 2007 for more).

^15^This is of course, not without a cost. Tuning takes time to affect the processing, and processing itself may also then take longer. That different visual tasks take different amounts of processing time is well documented and is related to dynamic tuning in Tsotsos et al. (2008), Tsotsos (2011). See also Figure 5 and caption.

^16^It should be noted that the original formulation included consideration of the set of world models *N* whose search efficiency can be logarithmically improved by hierarchical organization (Tsotsos, 1987). This is omitted here since it does not alter that nature of the problem.

^17^These predictions - all asserted before any supporting experimental data - include, for example, the suppressive surround in spatially attended stimuli, a suppressive surround in the attended feature dimension, the latency of attentional neural modulation having a top-down pattern, that neural modulation due to attention is present throughout the visual hierarchy, that neural baseline firing increases for an attended location and decreases elsewhere, and more.

^18^Amnon Sha'shua, for example, asserted this in his keynote lecture the 2016 IEEE Computer Vision and Pattern Recognition conference, Las Vegas NV (Sha'shua, 2016). Elon Musk also claimed autonomous driving is solved, for which vision is a key technology, in Eadicicco (2016).

^19^For example, the prediction of spatial surround suppression due to attention, first described in Tsotsos (1988b), was in fact “proved” infeasible in the brain by Crick and Koch (1990; p. 959) but now is widely confirmed (see review by Carrasco, 2011). See also the various peer commentaries published along with Tsotsos (1990).
